# Disruption of the Interaction between ORF33 and the Conserved Carboxyl-Terminus of ORF45 Abolishes Progeny Virion Production of Kaposi Sarcoma-Associated Herpesvirus

**DOI:** 10.3390/v13091828

**Published:** 2021-09-14

**Authors:** Joseph Gillen, Fanxiu Zhu

**Affiliations:** 1 Functional Cellular Networks Section, Laboratory of Immune System Biology, National Institute of Allergy and Infectious Diseases, National Institutes of Health, Bethesda, MD 20892-1892, USA; joseph.gillen@nih.gov; 2 Department of Biological Science, Florida State University, Tallahassee, FL 32306-4370, USA

**Keywords:** Kaposi sarcoma-associated herpesvirus (KSHV), tegument, ORF45, ORF33, TAT cell-penetrating peptide, viral assembly

## Abstract

The Open Reading Frame 45 (ORF45) of Kaposi sarcoma-associated herpesvirus (KSHV) is a gammaherpesvirus-specific, immediate-early, tegument protein required for efficient viral replication and virion production. We have previously shown that ORF45 interacts with the conserved herpesviral protein ORF33 through the highly conserved C-terminal 19 amino acids (C19) of ORF45. Because the deletion of C19 abolished ORF33 accumulation and viral production, we reasoned that this interaction could be critical for viral production and explored as an antiviral target for gammaherpesviruses. In work described in this article, we characterize this interaction in further detail, first by revealing that this interaction is conserved among gammaherpesviruses, then by identifying residues in C19 critical for its interaction with and stabilization of ORF33. More importantly, we show that disruption of the interaction, either by mutating key residues (W403A or W405A) in C19 or by using competing cell penetration peptide TAT-C19, dramatically reduce the yield of KSHV progeny viruses. Our results not only reveal critical roles of this interaction to viral production but also provide a proof of concept for targeting the ORF33-ORF45 interaction as a novel antiviral strategy against KSHV and other gammaherpesviruses.

## 1. Introduction

Kaposi sarcoma-associated herpesvirus (KSHV) is etiologically associated with Kaposi sarcoma (KS), primary effusion lymphoma (PEL), a subset of multicentric Castleman’s disease (MCD), and newly-defined KSHV inflammatory cytokine syndrome [1,2,3,4,5]. KSHV belongs to the Rhadinovirus genus (γ2) in the Gammaherpesvirinae subfamily which includes Rhesus rhadinovirus (RRV), herpesvirus saimiri (HVS), and murine gammaherpesvirus 68 (MHV-68). Among human herpesviruses, KSHV is closely related to Epstein–Barr virus (EBV) in the same Gammaherpesvirinae subfamily but different genus, Lymphocryptovirus (γ1) [6,7].

As a herpesvirus, KSHV has two distinct life cycles, known as latency and lytic replication. Latency is a quiescent state during which viral gene expression is limited to a few latent genes such as latency-associated nuclear antigen (LANA) and a dozen microRNAs. In contrast, lytic replication involves the expression of the full complement of viral genes that ultimately result in the production of progeny virions [8,9]. Like other herpesviruses, KSHV virions consist of four morphologically distinct structures: genome, capsid, tegument, and envelope. Among these, the tegument, which is located between the capsid and envelope, is the most complex in composition. Unlike the capsid proteins, which are well conserved across herpesvirus subfamilies, several prominent tegument proteins are unique to each subfamily [10,11,12,13,14,15].

Our interest is in ORF45, a multifunctional tegument protein that is found in only gammaherpesviruses. Although conserved, ORF45 homologues differ dramatically in protein length. KSHV ORF45 is the longest, at 407aa, while RRV, HVS, EBV, and MHV-68 have homologous proteins of 353, 257, 217, and 206 aa, respectively [16,17,18,19]. Partially complicated by the length variation, the overall sequence homology among these homologues is low. Only a few short, discrete regions can be aligned together. Among these regions, the extreme C-terminus has the highest homology, implying an important role for the conserved C-terminus. Indeed, we recently found that the C-terminal 19aa (C19) of KSHV ORF45 binds to ORF33 and helps to stabilize ORF33 in cells [20]. Importantly, we revealed that deletion of the C19 abolished the accumulation of ORF33 protein in cells and the production of progeny virions, confirming crucial roles of the ORF45 C-terminus as well as its interactions with ORF33 in the production of KSHV progeny virions [20].

Similar to ORF45, ORF33 is a tegument protein, but unlike ORF45 which is specific to gammaherpesviruses, ORF33 is conserved among all three herpesvirus subfamilies [11,21]. Its homologues, herpes simplex virus-1 (HSV-1, Alphaherpesvirinae), UL16, human cytomegalovirus (HCMV, Betaherpesvirinae), UL94, and Epstein–Barr virus (EBV) BGLF2 are all present in the tegument layer of mature virions [22,23,24,25], but the exact roles of the ORF33 homologues in herpesviral replication remain elusive. Deletion of HSV UL16 led to a moderate decrease in virion production [26] but the deletion of HCMV UL94 abolished the production of virions [23]. In gammaherpesviruses, ORF33 of MHV-68 was found to be essential because ORF33-null mutation abolishes the release of infectious virions but does not affect viral DNA replication, viral gene expression, or capsid assembly [27,28,29,30,31]. Our recent studies indicated that the deletion of ORF33 and/or its conserved partner, ORF38, reduced the production of progeny virions [21]. The defects appeared to be less severe than those caused by the deletion of the entire ORF45 coding sequence or by the deletion of the C19 [20,21]. A similar phenomenon was observed in MHV68 [29,30,32,33].

Because the C19 of ORF45 is sufficient and essential for binding to ORF33, and because the deletion of C19 is deleterious to the virus, we reasoned that this interaction could be critical for viral production and explored as an antiviral target for gammaherpesviruses. We, therefore, have characterized this interaction in further details. We first reveal that this interaction is conserved among gammaherpesviruses and then identify critical residues in C19 for its interaction with and stabilization of ORF33. More importantly, we show that the disruption of the interaction, either by mutating the key residues in C19 or by using competing cell penetration peptide TAT-C19, dramatically reduces the yield of KSHV progeny viruses. Our results not only reveal the critical roles of this interaction in viral production but also validate this interaction as a potential target for antiviral intervention of gammaherpesviruses.

## 2. Materials and Methods

### 2.1. Cell Cultures and Reagents

HEK293T cells were cultured in Dulbecco’s modified Eagle’s medium (DMEM) with 10% fetal bovine serum (FBS) and antibiotics. iSLK cells were cultured with the same medium plus 450 µg/mL G418 and 1 µg/mL puromycin [34]. The generations of iSLK.BAC16, iSLK.BAC16-ORF45 ΔC19, and iSLK.BAC16-ORF45 ΔC19R cells were described previously [20,35,36]. These cells were cultured similarly to iSLK with the addition of 400 µg/mL hygromycin B. Anti-HA, anti-FLAG M2, anti-GST antibodies, and EZview Red anti-FLAG M2 resin were purchased from Sigma-Aldrich (St. Louis, MO, USA). Anti-USP7 antibody was purchased from Bethyl Laboratories (Montgomery, TX, USA). Antibodies that detect ORF45 were generated as previously described [11,37]. Monoclonal antibodies against ORF26, ORF33, and ORF38 were generated as described [21]. Protein-G beads were purchased from Thermo Fisher Scientific (Waltham, MA, USA). EZ-Run Pre-Stained Protein Ladder was purchased from Fisher Scientific (Pittsburgh, PA, USA). TAT-C19 and TAT were synthesized by Biomatik (Wilmington, DE, USA).

### 2.2. Plasmid Constructs

Plasmids pCR3.1-ORF45, pCMV-3Tag1-KSHV-ORF33, pKH3-RRV-ORF45, pKH3-HVS-ORF45, pKH3-MHV68-ORF45, pKH3-EBV-BKRF4, pKH3-KSHV-ORF45, and derivatives have been described previously [16,37]. HSV-1 UL16 and EBV BGLF2 plasmids were provided by Dr. Jürgen Haas of the University of Edinburgh. The coding region of each viral gene was amplified by PCR then cloned into the pCMV-3Tag1 vector (Agilent Genomics, Santa Clara, CA, USA). HCMV UL94 plasmid was provided by Dr. Wade Bresnahan of the University of Minnesota, and the coding region was recloned into the pCMV-3Tag1 vector as well. RRV BAC genome was provided by Qiyi Tang of the Ponce School of Medicine and Health Sciences, and ORF45 was cloned using PCR then inserted into the pCMV-3Tag1 vector.

### 2.3. pGEX-5X Plasmid Constructs

To generate plasmids expressing the last 20 aa of ORF45 homologues fused to GST, we purchased synthesized oligonucleotides from Integrated DNA Technologies (sequences available upon request). For each homologue, the corresponding coding and anticoding strands with restriction digestion sites at the hanging ends were annealed and cloned into pGEX-5X (Pharmacia, now Cytiva, Marlborough, MA, USA). Mutant derivatives of each pGEX plasmid were generated as needed using the QuikChange protocol (Agilent Genomics, Santa Clara, CA, USA). Briefly, 125 ng each of sense and antisense primers encoding the desired mutation (primer sequences available upon request) were mixed with 10 ng of template, 1 × PFU enzyme buffer, 200 µM dNTP, and 1 µL PFU enzyme. Following PCR, 1 µL of DpnI enzyme (New England Biolabs, Ipswich, MA, USA) was added to each tube and the mix was then incubated at 37 °C for 1 h. Top10 *E.coli* cells were then transformed with 1 µL of the plasmid mixture and the resulting cells were grown overnight at 37 °C on LB-Amp plates. Positive clones were then isolated and the desired mutations were confirmed by sequencing.

### 2.4. Expression and Preparation of GST-Fusion Proteins

*E.coli* BL21 transformed with plasmids encoding GST or GST fusion proteins were induced with 1 mM isopropyl-β-D-thiogalactopyranoside (IPTG) for 3 h at room temperature. The cells were pelleted, washed once with PBS, and resuspended in PBS containing lysozyme and phenylmethylsulfonyl fluoride (PMSF). After sonicating twice, Triton X-100 was added to a final concentration of 1% and the mixture rotated at 4 °C for 1 h. After pelleting at 10,000× *g* for 10 min to remove cellular debris, the supernatant was incubated with glutathione agarose beads (Sigma-Aldrich Inc., St. Louis, MO, USA) at 4 °C overnight. After washing the beads 5 times with PBS, bound proteins were eluted using glutathione elution buffer (10 mM glutathione, 50 mM Tris-HCl, pH 8.5) and then dialyzed against PBS overnight. Concentrations were determined with a bicinchoninic acid protein (BCA) kit according to the manufacturer’s instructions (Pierce Biotechnology Inc., now part of Thermo Fisher Scientific, Waltham, MA, USA). The purity was assessed by SDS-PAGE followed by Coomassie Brilliant Blue staining.

### 2.5. Pull-Down Assays with GST Fusion Proteins

Twenty-four hours prior to lysing, HEK293T cells were transfected with pCMV-3Tag1-KSHV-ORF33 or its homologues, as indicated. After lysing, the cells in 1 mL of the whole-cell lysis buffer (50 mM Tris-HCl, pH 7.4, 150 mM NaCl, 1% Nonidet P-40, 1 mM sodium orthovanadate (Na_3_VO_4_), 40 mM glycerophosphate, 30 mM sodium fluoride, 10% glycerol, 5 mM EDTA, 1× protease inhibitor mixture (Roche, Indianapolis, IN, USA), and 1 mM PMSF) and pelleting the insoluble materials at 10,000× *g*, the supernatants were mixed with 20 μg of purified GST or GST fusion protein for 1 h at 4 °C. The mixture was then mixed with 50 μL of glutathione agarose beads and rotated for 1 h. The beads were pelleted by centrifugation at 8000× *g* for 1 min at 4 °C and washed 3 times using the whole-cell lysis buffer followed by using PBS twice. Finally, the beads were mixed with an equal volume of 2 × Laemmli loading buffer and boiled for 10 min before the associated proteins were resolved by electrophoresis on a 12% SDS-PAGE gel, at 110 V, for 90 min. The gels were then stained using Coomassie Brilliant Blue or analyzed by western blot for specific proteins.

### 2.6. Western Blot

Protein lysates were resolved by SDS-PAGE and transferred to nitrocellulose membranes. The membranes were blocked in 5% dried milk in 1× phosphate-buffered saline plus 0.2% Tween20 and then incubated with diluted primary antibodies for 2 h at room temperature or overnight at 4 °C. Anti-rabbit, anti-rat, or anti-mouse IgG antibodies conjugated to horseradish peroxidase (Pierce Biotechnology Inc., now part of Thermo Fisher Scientific, Waltham, MA, USA) were used as the secondary antibodies. An enhanced chemiluminescence system (Pierce Biotechnology Inc., now part of Thermo Fisher Scientific, Waltham, MA, USA) was used for detection.

### 2.7. ELISA with GST Fusion Proteins

Twenty-four hours prior to lysing, HEK293T cells were transfected with pCMV-3Tag1-KSHV-ORF33. After lysing the cells in 1 mL of whole-cell lysis buffer, and pelleting the insoluble materials at 10,000× *g*, the supernatants were pooled and stored on ice. Glutathione-coated 96-well plates (Pierce Biotechnology Inc., now part of Thermo Fisher Scientific, Waltham, MA, USA) were washed three times with 200 µL of PBST (PBS with 0.2% Tween20) per well. To each well, we then added 100 µL of GST-fusion-expressing *E.coli* lysate (prepared as per the purification protocol above) and rocked the plate at room temperature for 1 h. First, the wells were washed three times with 200 µL of PBST and then incubated with 100 µL of FLAG-ORF33-expressing cell lysate for 1 h at room temperature with rocking. Then, the wells were washed three times with 200 µL of PBST and then incubated with 100 µL of anti-FLAG IgG (1 µg/mL) in PBST + 3% BSA for 1 h at room temperature with rocking. Next, the wells were washed three times with 200 µL of PBST and then incubated with 100 µL of anti-mouse IgG-HRP (0.5 µg/mL) in PBST + 3% BSA for 1 h at room temperature with rocking. Finally, the wells were washed three times with 200 µL of PBST and then incubated with 100 µL of 1-Step Ultra TMB-ELISA substrate solution (Pierce Biotechnology Inc., now part of Thermo Fisher Scientific, Waltham, MA, USA) followed by reading on a spectrophotometer.

### 2.8. ORF33-Binding Assay

Twenty-four hours prior to lysing, HEK293T cells were transfected with pCMV-3Tag1-KSHV-ORF33, pCR3.1-KSHV-ORF45 wild type, or pCR3.1-KSHV-ORF45 mutants, as indicated. After lysing the cells in 1 mL of whole-cell lysis buffer, and pelleting the insoluble materials at 10,000× *g*, the supernatants were mixed to combine ORF33 with ORF45 wild-type or mutants for 1 h at 4 °C. The mixture was then incubated with 50 µL of EZview red anti-FLAG M2 affinity resin (Sigma-Aldrich Inc., St. Louis, MO, USA) for 1 h or overnight at 4 °C. After washing with the whole-cell lysis buffer and Tris-buffered saline (TBS: 50 mM Tris-HCl, pH 7.4, 150 mM NaCl), proteins were eluted by incubation with 150 ng/μL 3 × FLAG peptide in TBS for 1 h at 4 °C. The immunocomplexes were then analyzed by western blot.

### 2.9. Genetic Manipulation of KSHV BAC Genome

Mutagenesis of BAC16 was performed by using a recombineering system as previously described [20,21,38,39,40,41]. In brief, the Kan/I-SceI cassettes were amplified from plasmid pEPKan-S by PCR with primers as follow: ORF45-W403A-5′ (5′-GGCCTCCACGCCACCCCTGTGTGGAAACGGTGCATATAACGCGCCGTGGCTGGACTGATAAATAAGGATGACGACGATAAGTAGGG-3′) and ORF45-W403A-3′ (5′-TGTATTGACACCATTCTTTTATTTATCAGTCCAGCCACGGCGCGTTATATGCACCGTTTCCACAACCAATTAACCAATTCTGATTAG-3′) for the ORF45-W403A mutant, and ORF45-W405A-5′ (5′-CACGCCACCCCTGTGTGGAAACGGTGCATATAACTGGCCGGCGCTGGACTGATAAATAAAAGA AGGATGACGACGATAAGTAGGG-3′) and ORF45-W405A-3′ (5′-ATGGTCTGTATTGACACCATTCTTTTATTTATCAGTCCAGCGCCGGCCAGTTATATGCACCGTAACCAATTAACCAATTCTGATTAG-3′) for the ORF45-W405A mutant. The purified PCR fragment was electroporated into BAC16-containing GS1783 cells that had been induced at 42 °C for 15 min. The recombinant clones were selected at 32 °C on LB plates containing 34 µg/mL chloramphenicol and 50 µg/mL kanamycin and then characterized by RFLP (Restriction Fragment Length Polymorphism). Positive clones were induced at 42 °C again and plated on LB plates containing 1% L-arabinose for secondary recombination. Then, we picked replicates of the clones from L-arabinose plates onto plates with 34 µg/mL chloramphenicol alone, and plates with 34 µg/mL chloramphenicol plus 50 µg/mL kanamycin. The kanamycin-sensitive clones were second-recombinant clones and confirmed by RFLP and sequencing.

### 2.10. Quantification of Extracellular Virion Genomic DNA by Real-Time qPCR

Induced iSLK cells were analyzed for virion release into supernatants during the time course as previously described [16,20,21,35,41,42]. Viral DNA was isolated from the supernatant medium clarified by centrifugation at 1000× *g* for 10 min. One hundred µL of the supernatant was first treated with 10 U Turbo^TM^ DNase (Invitrogen, now part of Thermo Fisher Scientific, Waltham, MA, USA) in 1 × buffer at 37 °C for 1 h, incubated with 2 µL 0.5 M EDTA at room temperature for 5 min to inactivate DNase, then incubated with 20 µL proteinase K (Qiagen, Germantown, MD, USA) in 200 µL AL lysis buffer (Qiagen, Germantown, MD, USA) and 80 µL PBS buffer at 70 °C for 10 min. DNA was isolated by extraction in an equal volume of phenol-chloroform-isoamyl alcohol (25:24:1), precipitation in isopropanol with 6 µL 5 M NaCl and 2 µL 20 mg/mL glycogen, a cold 70% ethanol wash, and resuspension in 40 µL Tris-EDTA buffer (pH 8.0). Duplicate real-time PCRs in 20 µL volumes, containing 10% viral DNA, were analyzed by using SYBR green and specific primers for KSHV ORF73 (forward, 5′-CGCGAATACCGCTATGTACTCA-3′; reverse, 5-GGAACGCGCCTCATACGA-3′) [43] by CFX96 Real-Time System (Bio-Rad, Hercules, CA, USA). Viral copy numbers were analyzed with the CFX Manager (Bio-Rad, Hercules, CA, USA) and Microsoft Excel software calibrated to standards ranging from 1 to 10^7^ genome copies.

## 3. Results

### 3.1. The Carboxyl Terminus of ORF45 Is Highly Conserved among Gammaherpesviruses

**Figure 1:**
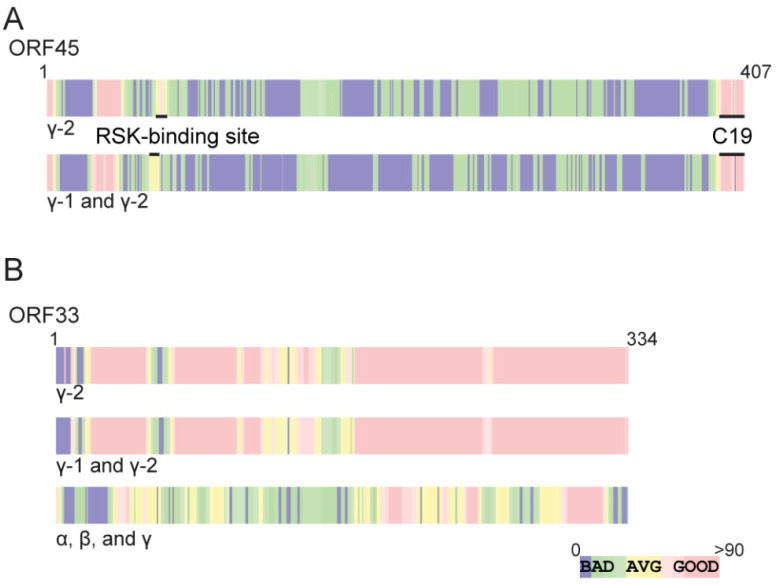
ORF45 is conserved among gammaherpesviruses with homology restricted mostly to the C-terminus while ORF33 is conserved among all herpesviruses. (**A**) ORF45 is conserved only among gammaherpesviruses, and high homology is limited to the C-terminal 19 aa (C19) region. T-Coffee alignment of ORF45 homologues of gamma-2 (γ-2) herpesviruses, or of both gamma-1 (γ-1) and γ-2 herpesviruses. (**B**) ORF33 is conserved among all three subfamilies of herpesviruses. T-Coffee alignments of ORF33 homologues of only γ-2 herpesviruses, or of both γ-1 and γ-2 herpesviruses. T-Coffee alignment of ORF33 homologues from alpha (α), beta (β), and γ herpesvirus shows sections of high and moderate homology throughout the coding region.

We collected protein sequences of ORF45 homologues of different gammaherpesviruses, including three γ-1 and sixteen γ-2 homologue sequences from the NCBI database. We aligned these sequences using the T-Coffee software package (http://www.tcoffee.org) [44]. This analysis revealed two short regions of ORF45 with noticeable conservation: the very end of the C-terminal ~19 aa that we have recently shown to bind to ORF33, and a region of no known function near the N-terminus. Interestingly, while C-terminal 19 is conserved among all members of the Gammaherpesvirinae subfamily, homology near the N-terminal is more limited (Figure 1A). A close inspection of the alignment revealed limited conservation of the crucial p90 RSK binding site motif around F66. Noticeably, this motif is shared only among γ-2 herpesviruses [35,45,46].

Similarly, we also compared protein sequences of ORF33 homologues. In stark contrast to the alignment of ORF45, significant homology among gammaherpesviruses was apparent across most of the coding region of ORF33 (Figure 1B). Unlike ORF45, which is unique to gammaherpesviruses, ORF33 is one of ~40 conserved proteins in all herpesvirus subfamilies [47]. When alpha and beta-human herpesviruses were included in the analysis, significant homology remained apparent in discrete regions, especially in the C-terminal half of the protein (Figure 1B). These analyses suggested that ORF33 is conserved among the entire herpesvirus family, but the overall sequence has diverged noticeably from alpha- and beta- herpesviruses.

### 3.2. Binding of the C-Terminus of ORF45 to ORF33 Is Conserved among Gammaherpesviruses

**Figure 2:**
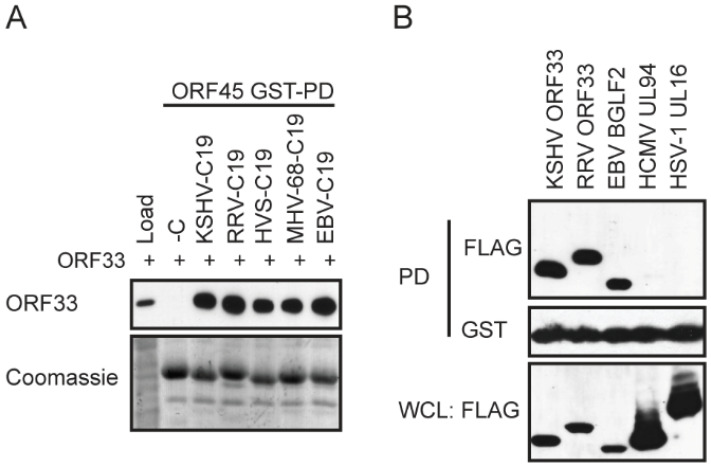
ORF45-ORF33 binding is conserved among gammaherpesviruses. (**A**) KSHV ORF33 binds to C19 of ORF45 homologues of other gammaherpesviruses. Lysates of FLAG-ORF33-expressing HEK293T cells were pulled down with purified GST-tagged fragments as indicated, and the eluates were analyzed by western blot. (**B**) KSHV ORF45-C19 binds only to ORF33 homologues of gammaherpesvirus. Lysates of FLAG-ORF33-homologue-expressing HEK293T cells were pulled down with purified GST-tagged KSHV ORF45-C19 and the eluates were analyzed by western blot.

Because the C-terminus of ORF45 that binds to ORF33 is highly conserved among gammaherpesviruses (Figure 1A), we were curious as to whether KSHV ORF33 can distinguish the C-terminus of different ORF45 homologues. We, therefore, examined whether the C-termini of different ORF45 homologues can also bind to KSHV ORF33, using GST pull-down assays. We fused the last C-terminal 19aa of ORF45 homologues of EBV (BKRF4), RRV, HVS, and MHV-68 to GST and used the fusion proteins to pull down FLAG-tagged KSHV ORF33. As shown in (Figure 2A), all of the ORF45 carboxyl tails bound to KSHV ORF33 equally well, suggesting that the C-termini of ORF45 homologues are indistinguishable to KSHV ORF33, at least in our experimental conditions.

Although ORF33 is conserved in all herpesviruses, the highest homology is shared among the gammaherpesviruses subfamily, and the overall sequence diverges from alpha- and beta- subfamilies. We next asked whether KSHV ORF45 binds to different ORF33 homologues of three subfamilies. Using GST pull-down assays, we found that KSHV ORF45 bound to ORF33 of gammaherpesviruses including KSHV ORF33, RRV ORF33, and EBV BGLF2, but not to UL16 of HSV-1 (alpha herpesvirus) or UL94 of HCMV (beta-herpesvirus) (Figure 2B). These results suggest that KSHV ORF45 is capable of binding to ORF33 homologues of gammaherpesviruses indistinguishably but is incapable of binding with ORF33 homologues of alpha- and beta- herpesviruses subfamilies. We, therefore, conclude that the ORF45-ORF33 interaction is conserved among the gammaherpesviruses subfamily.

### 3.3. Increase of ORF33 Stability by ORF45 via Their Interaction Is Conserved among Gammaherpesviruses

**Figure 3:**
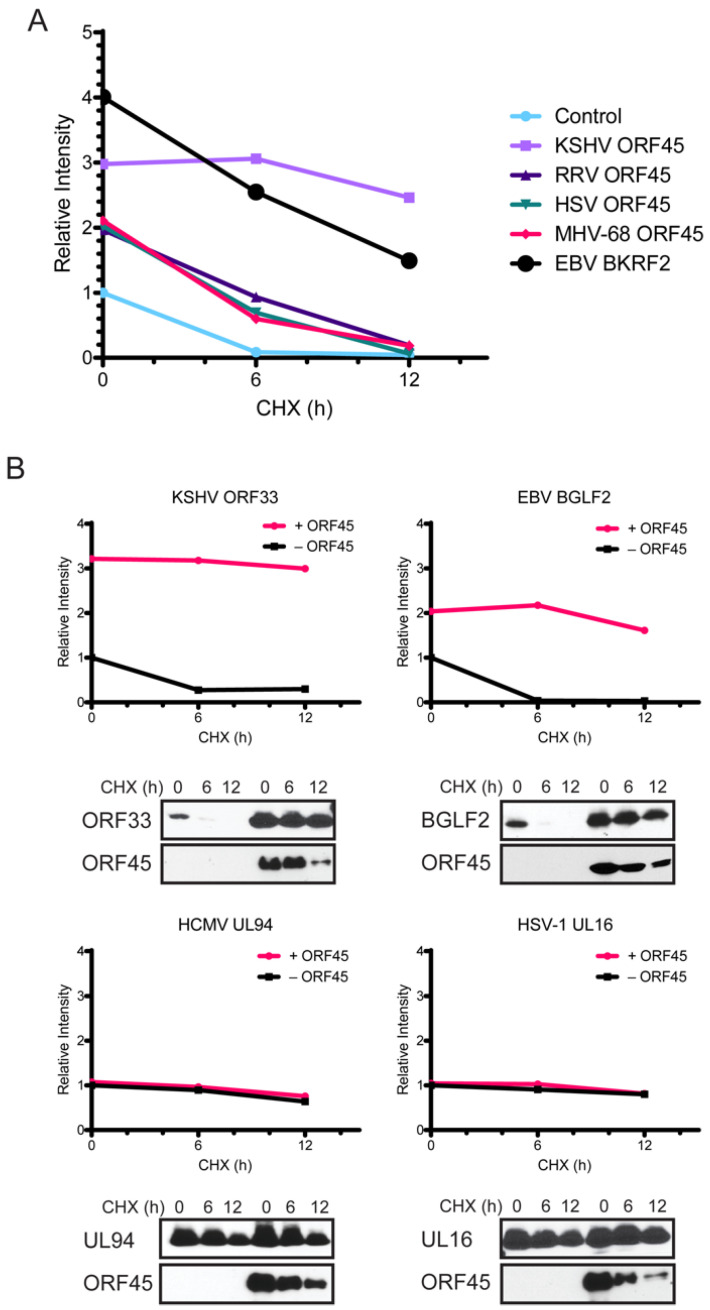
ORF45-induced stabilization of ORF33 is conserved among gammaherpesviruses. (**A**) Increase of KSHV ORF33 accumulation and stability by ORF45 homologues of gammaherpesviruses. HEK293T cells were transfected with KSHV ORF33 and homologues of KSHV ORF45 were then treated with cycloheximide (CHX) for the indicated time. The cells were lysed, and cell lysates were analyzed by western blot followed by ImageJ. (**B**) Only gammaherpesvirus ORF33 homologues showed increased stability in the presence of ORF45. ORF33 homologues of KSHV (γ-2), EBV (γ-1), HCMV (β), and HSV-1 (α) were transfected into HEK293T cells in the presence (circles) or absence (squares) of KSHV ORF45. The cells were treated with cycloheximide (CHX) for the indicated time. The cells were harvested and analyzed by western blot then ImageJ.

We have previously shown that ORF45 increases ORF33 stability in cells partially through their interactions [20]. Because the C-termini of gammaherpesviruses all bind to ORF33, we asked whether different ORF45 homologues also increase KSHV ORF33 stability in cells. We co-expressed KSHV ORF33 with KSHV ORF45 and its homologues of RRV, HVS, MHV-68, and EBV in HEK293T cells. We treated the cells with cycloheximide (CHX) to inhibit protein synthesis for different durations, and then examined ORF33 protein in cells by western blot. The relative levels of the proteins at each time point are presented in Figure 3A. In comparison to the empty vector control, all ORF45 homologues increased KSHV ORF33 stability to different extents, suggesting that the C-terminus of ORF45 contributes greatly to the enhanced stability of ORF33, though other regions may play additional roles.

We next examined whether KSHV ORF45 affects the stability of ORF33 homologues. We co-expressed KSHV ORF45 with FLAG-tagged ORF33 homologues from HSV-1, HCMV, KSHV, and EBV in HEK293T cells and performed analyses similar to the above. As shown in Figure 3B, ORF33 homologues of gammaherpesviruses were, overall, accumulated at lower levels than those of alpha- or beta- herpesvirus subfamilies in the absence of ORF45. Noticeably, co-expression of KSHV ORF45 increased the level of ORF33 homologues in gammaherpesviruses but had little effect on those of alpha- or beta- herpesviruses (Figure 3B). These results confirmed that the increase in ORF33 accumulation by ORF45 depends, at least partially, on their interactions, and that this property is conserved among gammaherpesviruses.

### 3.4. Two Conserved Tryptophan Residues in the C-terminus of ORF45 Are Critical for Its Binding to ORF33 and the Increase in ORF33 Stability

**Figure 4:**
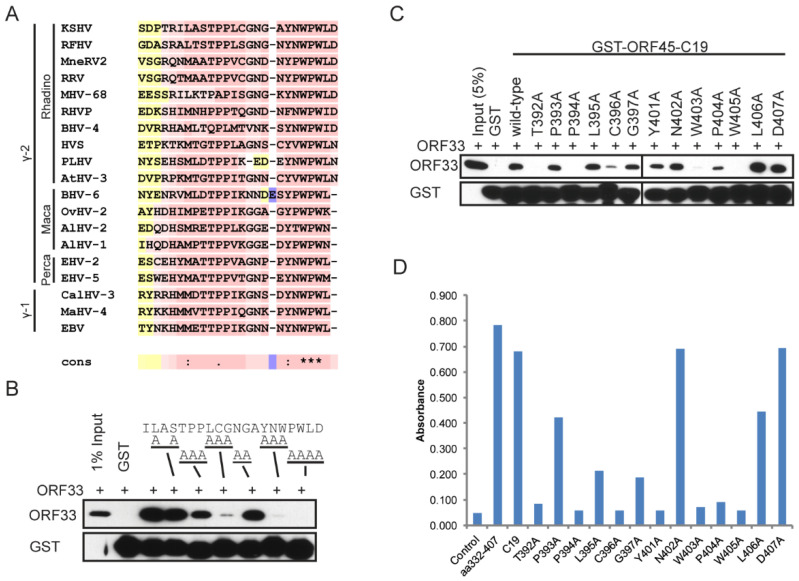
Identification of critical residues in ORF45 C19 region for binding with ORF33. (**A**) T-Coffee alignment of ORF45 C19 regions of gammaherpesviruses shows high conservation. (**B**) GST pull-down with triple alanine scanning mutants identifies residues that affect the binding of ORF33 to C19. Lysates of FLAG-ORF33-expressing HEK293T cells were pulled down with purified GST-tagged fragments as indicated and the eluates were analyzed by western blot. (**C**) GST pull-down with single alanine mutants identifies residues as critical for binding of C19 to ORF33. Lysates of FLAG-ORF33-expressing HEK293T cells were pulled down with purified GST-tagged fragments as indicated and the eluates were analyzed by western blot. (**D**) ELISA confirms the 7 residues in C19 as being required for binding of ORF45 to ORF33. Lysates of FLAG-ORF33-expressing HEK293T cells were incubated with GST-tagged ORF45 C19 fragments captured on a glutathione-coated 96-well plate. The bound ORF33 were detected by ELISA with anti-FLAG antibody.

A comparison of the C-terminus of ORF45 homologues revealed that the last 19aa region is highly conserved (Figure 4A). To reveal which residues are required for binding to ORF33, we generated a panel of alanine scanning mutants that changed every three or four amino acids to alanines (Figure 4B, top panel). Using GST-ORF45-C19 in pull-down assays, we identified several small regions including aa392–397 and aa401–407 as the most critical (Figure 4B, bottom panel).

To identify exact residues that are required for binding to ORF33, we further generated single alanine point mutants in the regions of aa392–397 and aa401–407 (Figure 4C). Using GST pull-down assays, we found that seven residues, T392, P394, C396, W403, P404, and W405, had the most dramatic effects (Figure 4C). To generate a more quantitative result, we used ELISA assays to measure the recovery of transfected ORF33 proteins from cellular lysates using 96-well plates that had been coated with glutathione and pretreated with GST-fusion constructs of the C19 region expressed in *E. coli* cells. These results confirmed the pull-down results and identified an additional residue (Y401) as critical (Figure 4D).

**Figure 5:**
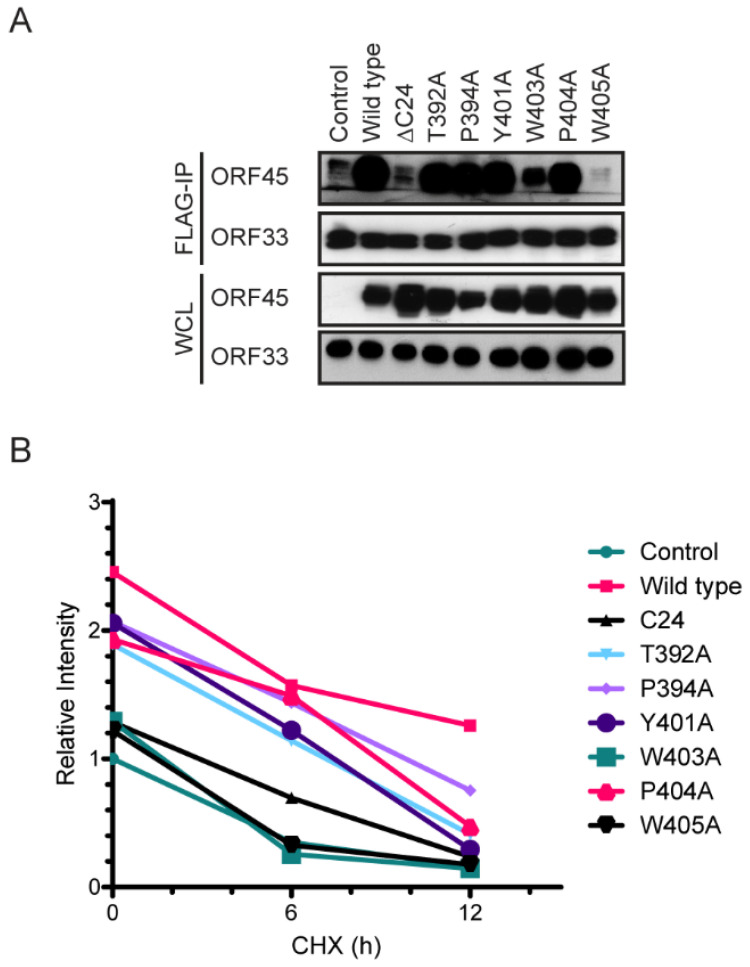
The invariably-conserved W403 and W405 of KSHV ORF45 are the most critical for binding with and stabilizing ORF33. (**A**) Mutation of either W403 or W405 in ORF45 abolishes binding to ORF33. Lysates of FLAG-ORF33-expressing HEK293T cells were mixed with lysates of HEK293T cells expressing either ORF45 wild-type or mutants, as indicated, and incubated for 1 h. The ORF33-bound proteins were immunoprecipitated with anti-FLAG M2 resin and analyzed by western blot. (**B**) Mutation of either W403 or W405 of KSHV ORF45 abolishes its ability to stabilize ORF33. HEK293T cells were transfected with FLAG-ORF33 with and without ORF45 wild-type or mutant, then treated with cycloheximide (CHX) for the indicated times. The cells were harvested and proteins in the lysates were analyzed by western blot then ImageJ.

We next introduced these mutations in the full-length ORF45 construct to examine how these mutations affect ORF45 binding to ORF33. Because the ORF45–ORF33 interaction affects the level of ORF33 if they are co-expressed in cells, we expressed ORF33 and ORF45 mutants in HEK293T cells separately. We next combined ORF45-expressing and ORF33-expressing cell lysates together. After 1 h of incubation, we precipitated FLAG-ORF33 and then probed the immunocomplexes for ORF45 by western blot (Figure 5A). The results revealed that the mutation of either W403 or W405 abolished ORF45 binding to ORF33. When we co-expressed ORF45 and ORF33, and then treated the cells with CHX for different durations, we found that W403A and W405A also failed to increase ORF33 stability (Figure 3B). These results suggest that W403 and W405, which are invariably conserved in all gammaherpesviruses, are the most crucial for binding to ORF33, and consequently, for the increase in ORF33 stability.

### 3.5. W403A or W405A Mutation in KSHV ORF45 Abolishes Production of Progeny Viruses

**Figure 6:**
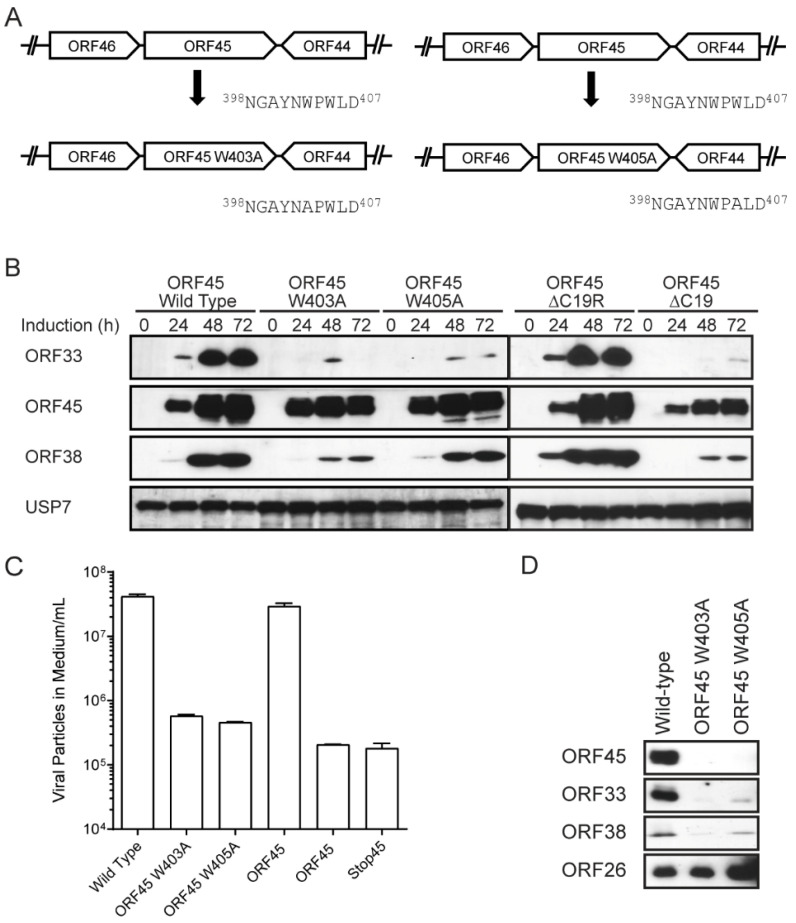
Binding of ORF33 to ORF45 is required for efficient lytic replication. (**A**) Diagram depicts W403A or W405A mutation in KSHV BAC16. (**B**) Mutation of either W403 or W405 abolishes accumulation of ORF33 in cells during lytic reactivation. Wild-type or mutant iSLK.BAC16 lines were induced with doxycycline and butyrate for the indicated times. The cells were harvested and analyzed by western blot. (**C**) Mutation of either W403 or W405 abolishes progeny virion production. Wild-type or mutant iSLK.BAC16 lines were induced for 120 h and the medium containing extracellular virions were collected. The medium was treated with DNase and capsid-protected DNA was used for qPCR to measure the viral genome copies. (**D**) Mutation of either W403 or W405 abolishes incorporation of ORF33 and ORF45 into extracellular virions. Wild-type or mutant iSLK.BAC16 lines were induced for 120 h and then the media were collected. The extracellular virions were concentrated by centrifugation and analyzed by western blot.

To determine how W403A and W405A affect KSHV lytic replication, we introduced the two mutations into BAC16 [36], an infectious bacterial artificial chromosome clone of the KSHV genome (Figure 6A). We transfected these BACs into iSLK cells and established stable cell lines, as described previously [16,20,21,35,42]. We treated the cells with doxycycline and sodium butyrate to induce KSHV lytic replication, and then monitored the viral protein expression by western blots (Figure 6B). We found that while both W403A and W405A were expressed at similar levels to the wild-type ORF45, the two mutations resulted in a dramatic decrease in the ORF33 protein level in cells. As controls, the deletion of the C19 or the entire ORF45 coding region also similarly reduced ORF33 accumulation, as we have previously reported [20].

We next determined the yield of progeny particles secreted in the culture medium by measuring the viral genome copy by qPCR, as described previously [16,20,21,35,42]. We found that W403A or W405A reduced the viral yield by ~50- and 100-fold, respectively, whereas the deletion of the C19 region or the entire ORF45 coding region resulted in over a 100-fold reduction in the viral yield. These results indicate that the ORF45–ORF33 interaction via the C-terminus of ORF45 is required for the production of KSHV progeny viruses.

### 3.6. W403A or W405A Mutation Dramatically Reduced Incorporation of ORF33 and ORF45 into Extracellular Viral Particles

We concentrated the extracellular viral particle through a 25% sucrose cushion as described previously [11,16,20,21,35,41,48]. After normalizing the number of virions based on viral genome copy numbers determined by qPCR, we analyzed virion protein by western blot (Figure 6D). Despite having capsid protein ORF26 at equivalent levels, both W403A and W405A mutant viruses contained little or no ORF33 or ORF45. The incorporation of ORF38, a conserved partner of ORF33 found in all three herpesvirus subfamilies, was also reduced. This experiment suggests that the disruption of the ORF45–ORF33 interaction is required for their own, and their partners’, incorporation into extracellular viral particles.

### 3.7. Synthetic TAT-C19 Peptide Interferes with ORF45–ORF33 Interaction and Inhibits Production of Progeny Viruses

**Figure 7:**
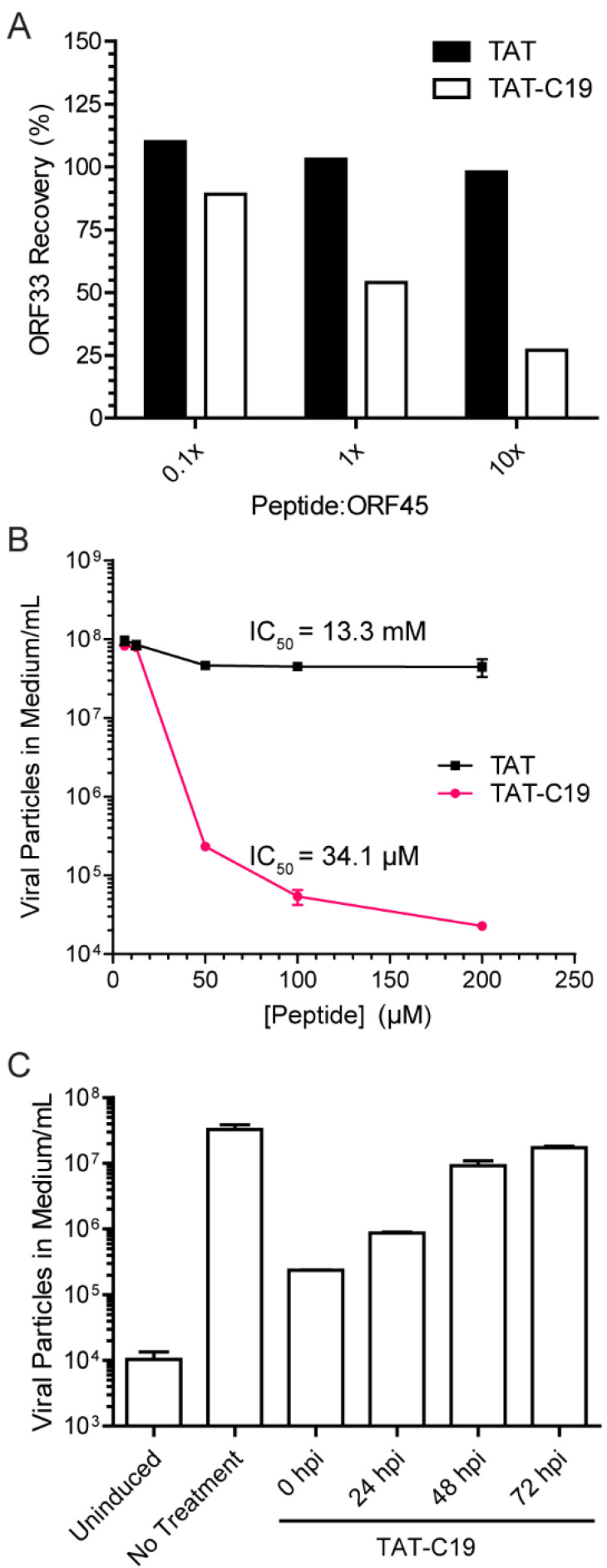
TAT-C19 peptide inhibits KSHV viral production. (**A**) TAT-C19 peptide inhibits ORF45–ORF33 interaction. ORF33 was mixed with GST-ORF45-332-407 in the presence of TAT or TAT-C19 at different ratios of peptide to GST-fused protein. The mixture was then added to a glutathione-coated 96-well plate and the recovery of ORF33 was measured by ELISA. (**B**) Treatment of iSLK.BAC16 cells with TAT-C19 inhibit virion production. Cells were treated with peptide and simultaneously induced by the addition of doxycycline and butyrate. After 5 days, the medium was collected and analyzed for viral genomes by qPCR. (**C**) Treatment with C19 is most effective early in the lytic cycle. Doxycycline and butyrate-induced iSLK.BAC16 cells were treated with 50 μM of TAT-C19 peptide at the indicated times post-induction, then the number of viral particles in the medium was measured by qPCR.

Because the last 19aa is sufficient for binding to ORF33 (Figure 2), we reasoned that a synthetic C19 peptide may compete with the full-length ORF45 for binding to ORF33. We, therefore, synthesized a TAT-C19 peptide in which the TAT sequence allows penetration into cells. To confirm this peptide indeed inhibits ORF45 binding to ORF33, we coated 96-well plates with ORF45 and incubated them with FLAG-ORF33 in the presence of TAT-C19 or TAT as the control. After washing, we measured the bound FLAG-ORF33 by ELISA. As shown in Figure 7A, the TAT-C19 peptide, but not the TAT control peptide, inhibited ORF33 binding in a dose-dependent manner.

To determine whether the TAT-C19 peptide can inhibit KSHV lytic replication, we treated iSLK.BAC16 cells with TAT-C19 and the control TAT peptides. We found a dose-dependent reduction of the viral yield by TAT-C19, by three logs of magnitude at the highest concentration of 200 µM. We estimated the IC_50_ to be ~34.1 µM (95% confidence interval of 28.17–41.25 µM). In contrast, treatment with TAT had little effect (IC_50_ of 13.3 mM, 95% confidence interval of 0.695–253 mM) (Figure 7B). To determine the most effective time at which peptide administration inhibited viral particle production, we induced the lytic cycle in iSLK.BAC16 cells and then treated the cells with 50 µM of TAT-C19 at 0, 24, 48, and 72 h post-induction (hpi) (Figure 7C). While all treatments decreased the viral particle output, we found that the most significant effects were seen if the cells were treated between 0 and 24 hpi. These results indicate that the peptide was most effective early in lytic reactivation. The effect was not caused by cytotoxicity because the treatment of SLK cells with TAT-C19 had no apparent effect on SLK cell viability up to 48 h (data not shown). This experiment suggested that the disruption of the ORF45–ORF33 interaction inhibits the production of KSHV progenies.

## 4. Discussion

Although ORF33 homologues are found in all herpesviruses, divergence is noticeable among three subfamilies. Unlike ORF33, ORF45 is unique to gammaherpesviruses, with no homologues in alpha- or beta- herpesviruses. Although conserved, the homology among ORF45 homologues is limited to mostly the C-terminal end (Figure 1). We showed that KSHV ORF45 interacts with ORF33 homologues of gammaherpesviruses indistinguishably but fails to bind to UL16 of the alphaherpesvirus HSV-1 or UL94 of the betaherpesvirus HCMV, suggesting that the ORF33–ORF45 interaction is conserved among gammaherpesviruses. This observation is consistent with the fact that ORF45 is a gamma subfamily-specific tegument protein (Figure 2). Concurrent with their interaction, ORF45-induced stabilization of ORF33 is also conserved among gammaherpesviruses (Figure 3). We revealed the two invariably conserved tryptophan residues in C19 to be the most critical for the interaction with ORF33 (Figure 4 and Figure 5). Change of the two residues (W403A and W405A) not only compromised ORF45′s ability to stabilize ORF33 but also abolished viral production. The mutation also decreased the incorporation of ORF45 and ORF33 as well as other tegument proteins into progeny virions. These results suggest that the ORF33–C19 interaction is required for the production of progeny virions of KSHV and for the assembly of tegument proteins into virions. More importantly, we showed that a synthetic C19 peptide exhibited a dominant negative effect and dramatically reduced the KSHV viral yield, suggesting that the disruption of the ORF33–ORF45 interaction effectively blocks KSHV viral production. Taken together, these results demonstrate that the interaction between ORF33 and ORF45 via C19 is crucial for the production of KSHV progeny virions. Moreover, our work has established a proof of concept for targeting the ORF33–ORF45 interaction for a novel antiviral strategy against KSHV. Because of the conservation of the ORF33–ORF45 interaction, this strategy should be applicable to other human and animal gammaherpesviruses including EBV as well as bovine and equine herpesviruses.

Although the ORF45–ORF33 interaction is important for KSHV replication, it remains unclear how this interaction is involved in KSHV replication. Our results showed that the ORF45–ORF33 interaction is required for the accumulation of the ORF33 protein in cells. A reasonable hypothesis is that the stoichiometric interactions between ORF33 and other virion proteins need to be maintained for optimal viral assembly. ORF33 is known to interact with ORF38 and this interaction appears to be conserved among all three subfamilies of herpesviruses [21,23,49,50,51,52,53]. In addition, a recent report indicates that ORF33 binds to capsids directly in the nucleus prior to primary envelopment [29]. When expressed alone, ORF33 and its homologues of the gammaherpesvirus were much less stable than their homologues of alpha- and beta- herpesviruses (HSV-1 UL16 and HCMV UL94, respectively). Conceivably, the increase of ORF33 accumulation by ORF45 is crucial for its function in KSHV lytic replication. We found that ORF45-induced stabilization in ORF33 is conserved among gammaherpesviruses: namely, KSHV ORF45 increased the stability in ORF33 homologues of other gammaherpesviruses, and conversely, ORF45 homologues of other gammaherpesviruses also increased the stability in KSHV ORF33. All examined ORF45 homologues increased the accumulation of KSHV ORF33 but to different extents: KSHV and EBV did better than RRV, HVS, and MHV-68 (Figure 3A). This is not surprising because we have previously shown that additional mechanisms other than the ORF33–ORF45 interaction via C19 are involved. Specifically, we showed that the recruitment of USP7 also contributed to the increase in ORF33 stability by KSHV ORF45 [20]. Curiously, however, other homologues including EBV BKRV4 seem to lack the USP7 binding motif. The strong increase in KSHV ORF33 accumulation by EBV BKRF4 suggests other mechanisms are involved. One possibility is the localization of the ORF45 homologue. Both KSHV and EBV localize to both the nucleus and cytoplasm of transfected cells while the RRV, HVS, and MHV-68 homologues localize exclusively to the nucleus [16]. Overall, the mechanisms by which ORF45 stabilizes ORF33 seem to be complex and may involve additional associated proteins and/or regulation of subcellular localization. Because ORF33 is intrinsically unstable and its accumulation in cells depends strictly on its interaction with ORF45, we were unable to distinguish the impacts of the loss of the ORF45–ORF33 interaction from those of the loss of the ORF33 protein level in cells. The generation of ORF33 mutants with increased protein stability and/or with a deficiency in binding to ORF45 would be valuable for further mechanistic studies. However, our repeated attempts to map domains of ORF33 affecting its stability or critical residues for its interaction with ORF45 have so far been unsuccessful.

We, and others, have reported that the deletion of ORF33 resulted in the reduction in the release of extracellular virions of KSHV, MHV-68, and EBV with minimal impact on the viral genome replication or gene expression [21,29,30,50]. The deletion of KSHV ORF45, or the homologues in EBV, RRV, and MHV-68, also reduced the yield of extracellular viruses [17,18,19,32,33]. A possible function of ORF33 and ORF45 could be to assist in the assembly of the progeny virions. Consistent with this model, the deletion of ORF33 or its homologues has been shown to interfere with egress and viral particle assembly [30,50,54]. ORF45 has been found to bind the kinesin II motor protein KIF3A in a mechanism that is hypothesized to transport capsids from the nucleus to the envelopment sites found in the cytoplasm [55]. More recent work reinforces the roles of ORF45 in viral assembly [56,57]. We speculated that the binding of ORF33 and ORF45 is required for the transportation of capsids through the cytoplasm, a model that has also been proposed in the related murine herpesvirus MHV-68 [29,30,33]. The transportation of capsids through the cytoplasm is theorized to begin at the nuclear membrane following the envelopment/de-envelopment of the genome-packed capsids [58]. Recently, the binding of ORF33 to the capsids of MHV-68 particles was shown to occur in the nucleus of infected cells prior to the primary envelopment at the nuclear membrane [29]. It will be interesting to know where the ORF45–ORF33 interaction first occurs and whether ORF45 is assembled into virions in the nucleus or cytoplasm. Although KSHV ORF45 is a mostly cytoplasmic protein, while most of the homologues of other gammaherpesviruses are typically nucleic, we have shown that KSHV ORF45 shuttles between nucleus and cytoplasm and that a conserved function in the nucleus is important for progeny viral production [16]. We observed that KSHV ORF45 and ORF33 colocalize in both the nucleus and cytoplasm [20]. Given a potential role of the ORF33–ORF45 interaction in capsid maturation and egress, the presence of ORF33 and ORF45 in the nucleus could act to facilitate the egress of capsids from the nucleus to the sites of secondary envelopment. These results together suggest that the ORF45–ORF33 interaction may be an early event in the assembly of infectious virions. The disruption of this interaction may, therefore, represent an effective antiviral strategy against gammaherpesviruses.

We noticed that not all ORF45 in cells bind to ORF33 [20]. We speculate that binding to ORF33 is required for assembly of ORF45 into the tegument and subsequent export of virions. It is unclear whether the C19 itself serves as a packaging signal for ORF45 into virions, and whether it is sufficient for the assembly of heterogeneous fused protein into viral particles. We have recently shown that the loss of either, or both, ORF33 and ORF38 produced similar phenotypes with a ~10-fold decrease of viral yield and reduced incorporation of ORF45 into virions. We noticed that the loss of ORF45, the deletion of ORF45 C19, or W403A/W405A mutations, resulted in more severe defects in virion production than the loss of either, or both, ORF33 and ORF38. This observation suggests that the interaction with ORF33 is not the only function encoded by the C19 region. Further research is needed to reveal additional functions of the conserved C19 region.

The stark differences between ORF33 and ORF45 conservation are of great interest. Using T-Coffee [44], we compared gammaherpesvirus homologues of all reported tegument proteins in the NCBI database. We found that nearly all exhibit high conservation (average T-Coffee scores of 85.3 with a standard deviation of 6.8). We also analyzed the variability of protein length among homologues of the known tegument proteins and found most differ minimally (average length variability was 24% with a standard deviation of 19%). Of all known tegument proteins, ORF45 has the lowest degree of conservation with a T-Coffee score of 68.1 in comparison to an average of 85.3 ± 6.8, and the highest protein length variability of 80% in compassion to an average of 24% ± 19%. In contrast, ORF33 is highly conserved, with a T-Coffee score of 89.9 and a length variability of 5%. Despite the tremendous divergence of ORF45 protein length and amino acid sequence, the C19 region remains highly conserved, implying its crucial roles for the viruses. Our work revealed that the C19 region, via interaction with ORF33, is critical for viral production and that targeted disruption of this interaction can be an effective antiviral approach against gammaherpesviruses.

## Data Availability

Not applicable.
